# Mid-infrared spectroscopy dataset of glass with tholeiitic, boninitic, and komatiitic compositions

**DOI:** 10.1016/j.dib.2026.113378

**Published:** 2026-10-23

**Authors:** Andreas Morlok, Oliver Burghardt, Stephan Klemme, Iris Weber, Maximilian P. Reitze, Harald Hiesinger

**Affiliations:** a Universität Münster, Institut für Planetologie, Wilhelm-Klemm-Strasse 10, 48149, Münster, Germany; b Universität Münster, Institut für Mineralogie, Correnstrasse 24, 48149, Münster, Germany

**Keywords:** Ftir spectroscopy, Remote sensing, Minerals, Earth, Mercury

## Abstract

Mid-infrared diffuse reflectance spectra of sieved size fractions are provided (0 µm - 25 µm, 25 µm - 63 µm, 63 µm - 125 µm and 125 µm - 250 µm) from 2 µm to 18 µm obtained from synthetic glass analogs for boninite, komatiites, and basalt.

The synthetic glass with tholeiitic basalt composition shows the Christiansen Feature (CF) at 8.3 µm and a single strong Reststrahlenband (RB) at 10 µm to 10.1 µm. The boninite glasses have the CF at 7.9 µm to 8 µm and the dominating RB at 9.5 µm to 9.6 µm, at 9.7 µm to 10 µm and 10.6 µm to 10.8 µm, respectively. The komatiitic glass exhibit clear crystalline features between 10.1 µm and 10.2 µm, 10.7 µm and 10.9 µm, and 11.2 µm to 11.3 µm. A Transparency Feature (TF) is at 12.2 µm and 12.3 µm.

Together these spectra provide a spectral reference for interpreting mid-infrared analyses from terrestrial and planetary surfaces.

Specifications Table

**Table:** 

Subject	Earth & Environmental Sciences
Specific subject area	*A reference mid-IR dataset of synthetic glass analogs with basaltic, komatiitic, and boninitic compositions.*
Type of data	*Table, Image, Raw Spectra*
Data collection	*Samples were synthesized using reagent-grade oxides, which were melted in air at 1400* °*C. Mid-Infrared diffuse reflectance spectra were obtained of sieved size fractions (0 µm - 25 µm, 25 µm - 63 µm, 63 µm - 125 µm and 125 µm - 250 µm), using a Vertex 70v FTIR spectrometer at the Institut für Planetologie (Universität Münster) Measurements were made using a liquid nitrogen cooled MCT-detector from 2* *µm to 18* *µm at low pressure (10^−3^* *bar). For calibration, diffuse gold standard was applied. For spectral quality, always 512 scans were measured.* *Chemical compositions and visual data were obtained using a JEOL 6610 scanning electron microscope (SEM) with an electron dispersive X-ray spectrometer (EDX) at the Institute of Mineralogy in Münster. Backscattered electron (BSE) images were acquired at an acceleration voltage of 20* *kV. For SEM/EDX analyses, natural mineral standards were used.*
Data source location	*Synthetic glass samples made at Universität Münster, Institute for Mineralogy, Correnstrasse 24, 48,149 Münster, Germany..*
Data accessibility	*The spectra presented are immediately available at the ZENODO repository, and after publication also in the IRIS database of the Institut für Planetologie in Münster* Repository name: ZENODO Data identification number: 10.5281/zenodo.18632810 Direct URL to data: https://doi.org/10.5281/zenodo.18632810 Instructions for accessing these data: All data are downloadable as single files from the repository under the DOI.
Related research article	*n/a*

## 1 Value of the Data

• Komatiites and Boninites are important Mg-rich rocks that formed by high degrees of melting, and especially komatiites were mainly formed during the evolution of early Earth. The latter were formed under conditions that differ significantly different from today’s terrestrial environment; therefore, fresh samples are rare [[1], [2], [3], [4], [5], [6], [7]]. Furthermore, there is indication that similar rock types occur on the surface of other terrestrial planets such as Mercury [e.g., [8], [9]]
• One of the techniques to identify the nature of volcanic rocks on the surface of planetary bodies is mid-infrared spectroscopy [10] by remote sensing, e.g., the MERTIS instrument on-board the BepiColombo mission on its way to Mercury [10]. However, the interpretation of remote sensing mid-infrared data requires laboratory-generated spectra of well-defined analog materials. Ideally suited for this purpose are tailor-made synthetic materials consisting either of minerals, rocks or glasses, especially relevant for rock types that are difficult to acquire in unaltered or non-weathered states as is the case of the komatiites and boninites [e.g., 3,7,10,11].
• Hence, the mid-infrared reflectance spectra of synthetic glass analogs with komatiitic, boninitic, and basaltic compositions are provided. These glass compositions are based on terrestrial rock compositions but they are also similar to those observed for the surface of Mercury. The mid-IR spectra of these samples fill a significant gap in existing spectral databases [[8], [9], [10], [11], [12]] of mid-IR spectra for space science especially for the MERTIS instrument on BepiColombo [10,11].

## 2 Background

Partial melting of peridotite mantle leads to the formation of primary melts. The chemical compositions of these partial melts depend strongly on temperature, pressure, and redox conditions. Whilst lower degrees of melting leads to basalts, high degrees of melting leads to more Mg-rich komatiites and boninites, the latter of which are important rock types related to processes on the very young Earth but they have also been reported on other terrestrial planets. Chemical and genetic affinities between komatiites, boninites, and the common tholeiitic basalts warrant systematic data of these materials [e.g. 3,4].

Mid-infrared spectroscopy is a technique that is commonly employed to characterize the composition of planetary surface with remote sensing [10,11]. Mid-infrared-spectra of laboratory-made glasses with boninitic and komatiitic compositions are provided based on the fact that regions on the surface of Mercury are thought to contain similar rock types [8,9]. Given the environmental conditions on the hermean surface, such as high contents of impacts melts, glasses are expected to be a major component of the surface regolith [10,13]. The mid IR range was investigated corresponding to the range of the MERTIS instrument on BepiColombo that is on its way to Mercury [10] and this will allow mapping the surface of this planet with high spatial resolution.

**Boninites** on Earth are rare, primitive volcanic rocks with high SiO_2_ (>52%wt.), MgO (> 8 wt%), and low-TiO_2_ (< 0. 5wt%) contents [1]. They generally contain olivine phenocrysts, orthopyroxene, and clinopyroxenes and tend to be glass rich (30% - 50%) [6]. Boninites form on Earth through partial melting of depleted mantle peridotite at relatively shallow depth (< 50 km) and high temperature (up to 1350 °C), typically associated with subduction zone settings and are thus an important marker for tectonic processes (e.g., subduction initiation) especially in early Earth and potentially other planets [2,3]. Alternatively, boninites may also originate from plume-sourced magmas in Archean settings [3]. Several terrains with Boninitic-like composition have been identified on Mercury based on their chemistry provided by MESSENGER [8,9,12]. Hermean boninites are very low in Fe and extremely rich in Na compared to boninites found on Earth and are therefore referred to as ‘alkali-rich boninites’ [8,9,12]. Phase equilibrium experiments suggest that H_2_O is not required to produce boninitic melts on Mercury, generated by dry melting of a reduced (ΔIW: 0 to −7) hermean mantle at low-pressures [9].

Terrestrial **komatiites** are ultramafic volcanic rocks with an MgO content higher than 18% [1]. They are typically formed within the Archean (> 2.7 Ga), and constitute the oldest lavas known – but rare younger Palaeozoic and Mesozoic komatiites do also exist [1]. Because of the age, almost all terrestrial samples are often metamorphosed and heavily altered [7].

Komatiitic melts have very low viscosities and they erupted at very high temperatures (>1400 °C). They formed on Earth by high-degree partial melting of the mantle due to a much higher mantle temperature in the Archean (for a review see e.g. [4]). Komatiite rocks in the Archean have also been proposed as a setting for the formation of life (e.g., the relation to high nickel sulphide deposits) and are thus of astrobiological interest (see discussion in [4]).

Our basaltic glass is based on a terrestrial **tholeiitic basalt**. These are formed by melting by shallow melting of peridotite, typical for the ocean island and mid-ocean ridge settings. This composition serves as analogue for the more common terrestrial basalts.

## 3 Data Description

The tholeiitic basaltic glass 674 Tholeiitic Basalt exhibits 13.4 wt% Al_2_O_3_ and 9.6 wt% MgO (Fig. 2, Table 1). The infrared spectrum shows no crystalline RBs, and is dominated by a single amorphous band at 9.98 µm – 10.05 µm. The CF is at 8.28 µm to 8.31 µm (Fig. 1, Table 2).

**Table 1:** Chemistry of the finished analog samples Sources: [5–7], obtained by SEM/EDX (in wt %). Detection limit for each element is 0.1 wt%. n.d. = Not detected. The numbers on top are the ID number entries in the IRIS database where the data are available [11].

	674 Tholeiitic Basalt	677 Boninite	676 Boninite variation	678 Komatiite	675 Komatiite Mg-rich
**Na_2_O**	3.5	1.4	1.5	0.9	0.4
**MgO**	9.6	12.3	17.5	20.7	30.7
**Al_2_O_3_**	13.4	11.3	11.3	10.7	7.5
**SiO_2_**	45.5	56.8	57.0	45.5	49.5
**P_2_O_5_**	0.6	0.1	0.1	n.d.	n.d.
**SO_3_**	n.d.	n.d.	n.d.	n.d.	n.d.
**K_2_O**	1.7	0.5	0.1	0.1	0.2
**CaO**	9.2	7.8	2.6	9.3	5.5
**TiO_2_**	3.3	0.3	0.3	0.7	0.1
**Cr_2_O_3_**	n.d.	n.d.	0.1	n.d.	n.d.
**MnO**	n.d.	0.1	0.1	n.d.	0.1
**FeO**	12.7	9.4	9.6	12.2	6.1
**NiO**	n.d.	n.d.	n.d.	n.d.	n.d.
**SUM**	99.5	100.0	100.2	100.0	100.1

**Fig. 1:**
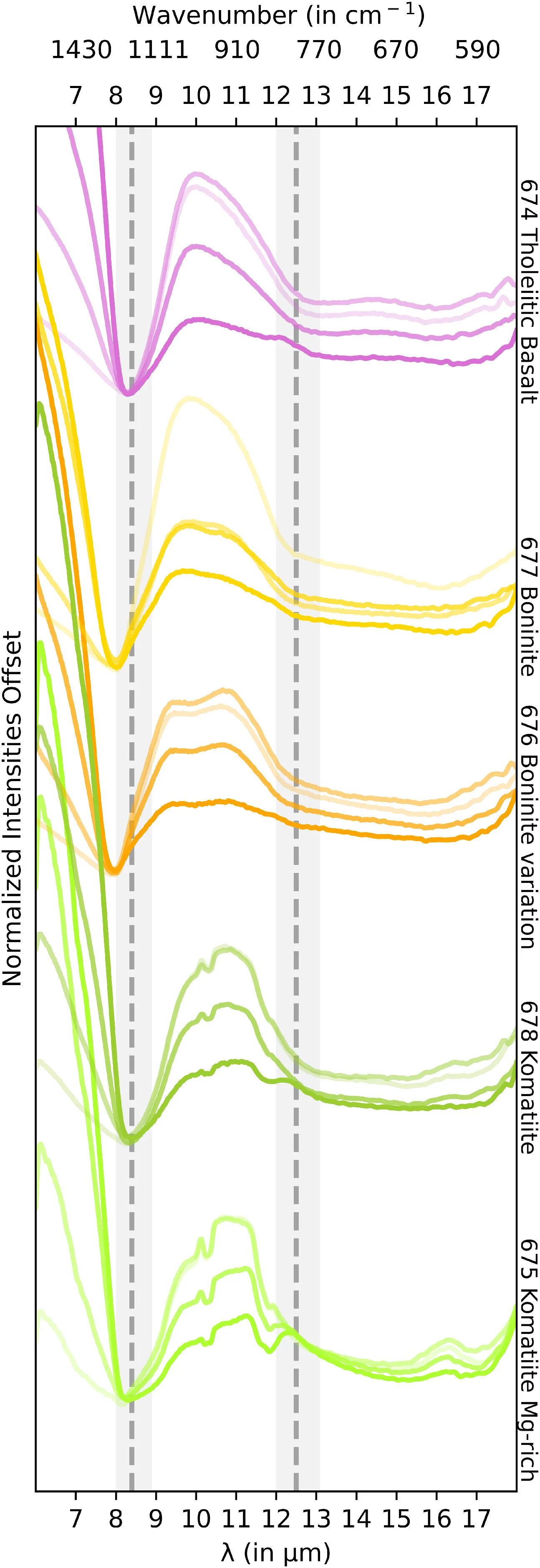
Spectra of all four size fractions of the five synthesized samples (wavelength in µm). Finest size fraction (0 – 25 µm) in full color, coarsest fraction (125 – 250 µm) in lightest shade.

**Table 2:** Band position of diffuse reflectance spectra of the four size fractions for each sample. CF=Christiansen Feature, TF: Transparency Feature, both are in µm. Standard error is ±0.02 µm for each band position.

		CF				TF			
674 Tholeiitic Basalt 0–25		8.31		10.05					
674 Tholeiitic Basalt 25–63		8.31		10.02			14.21		
674 Tholeiitic Basalt 63–125		8.31		9.98			14.57		
674 Tholeiitic Basalt 125–250		8.28		10.00			14.48		
677 Boninite 0–25		8.02	9.69						
677 Boninite 25–63		8.02	9.79						
677 Boninite 63–125		8.00	9.96						15.80
677 Boninite 125–250		8.04	9.81						
676 Boninite variation 0–25		7.98		10.82					
676 Boninite variation 25–63		7.93	9.47	10.69					
676 Boninite variation 63–125		7.91	9.59	10.69					
676 Boninite variation 125–250		8.00	9.47	10.64					
678 Komatiite 0–25		8.41				11.15			
678 Komatiite 25–63		8.35	10.16	10.82					
678 Komatiite 63–125		8.24	10.16	10.87					
678 Komatiite 125–250		8.26	10.16	10.74					
675 Komatiite Mg-rich 0–25		8.32	10.13		11.29	12.30		16.24	
675 Komatiite Mg-rich 25–63		8.24	10.13		11.23	12.17		16.30	
675 Komatiite Mg-rich 63–125		8.18	10.13	10.77				16.36	
675 Komatiite Mg-rich 125–250		8.15	10.13	10.77				16.36	

The average boninitic composition observed in [5,6] was used as a base for the composition of the (Fig. 2, Table 1). 677 Boninite shows spectral features of glassy materials: a single, broad feature between 8 µm and 12 µm [14,15], with a peak between 9.69 µm and 9.96 µm. The CF is at 8 µm – 8.04 µm, with no clear TF recognizable (Fig. 1, Table 2).

**Fig. 2:**
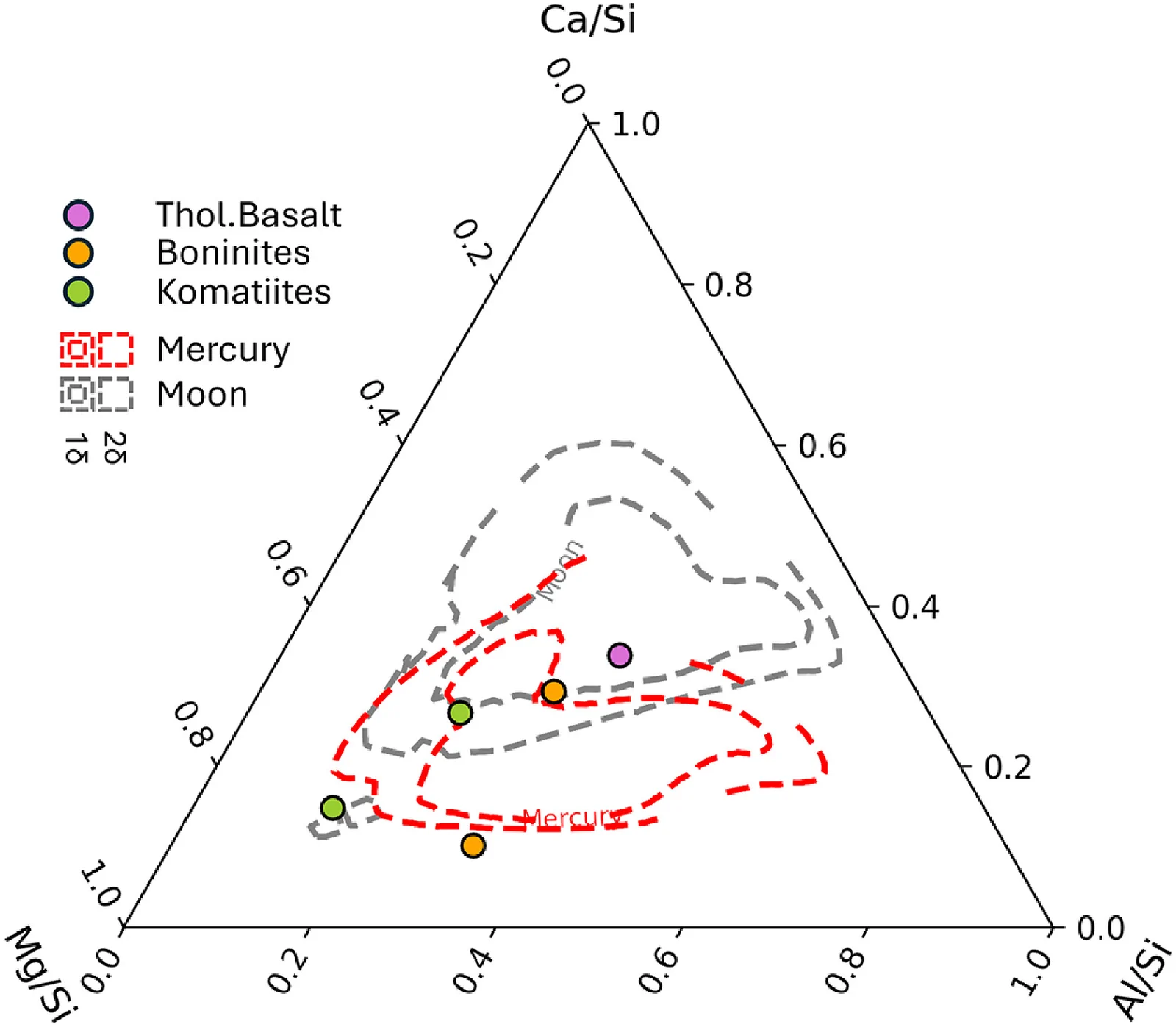
Chemical composition of the glasses, given as elemental ratios Mg/Si, Ca/Si, Al/Si, calculated from the SEM/EDX oxide wt.% in Table1. Synthesized samples: Green: Komatiites, Orange: Boninites, Pink: Tholeiitic Basalt. For comparison, the composition of synthetic analog materials with the chemical composition of surface regions of Mercury (red) and the Moon (grey) is shown with dashed lines: (one and two sigma). Sources literature data: [13].

The second boninitic glass (676 Boninite variation) was produced to investigate the impact of increased MgO. It contains 2.6 wt% CaO and 17.5 wt% MgO (Fig. 2, Table 1). The spectrum of 676 Boninite variant is different from 677 Boninite: the main RB is divided into two features – a clear peak at 10.64 µm to 10.82 µm, and a second one at 9.47 µm to 9.59 µm. The CF is slightly shifted – from 7.91 µm to 8 µm, with no clear TF visible (Fig. 1, Table 2).

Two distinct komatiitic compositions were chosen, they are based on terrestrial rock samples covering the chemical range described in [7]. The first komatiitic glass (675 Komatiite Mg-rich) is rich in MgO (30.6 wt%) and low in TiO_2_ (0.1 wt%) and FeO (6.1 wt%) (Fig. 2, Table 1). The spectra show strong crystalline RB of at 10.13 µm, 10.77 µm and 11.23 µm to 11.29 µm. Also, a TF is visible at 12.17 µm to 12.3 µm. The CF is between 8.15 µm and 8.32 µm (Fig. 2, Table 2).

The second composition (678 Komatiite) is lower in MgO (20.7 wt%) and higher in FeO (12.2 wt%) and TiO_2_ (0.7 wt%) (Fig. 2, Table 1). The spectrum also shows significant RBs at 10.16 µm, 10.74 µm to 10.87 µm and 11.15 µm, but clearly weaker that for 675 Komatiite Mg-rich. A weak TF is observed at about 12.3 µm. The CF is between 8.24 µm and 8.41 µm (Fig. 1, Table 2).

*SEM images for both the boninites and the tholeiitic basalts (* Fig. 3 *) display highly homogenous materials with no discernible crystals, contrary to the komatiites, which contain ample crystals.*

**Fig. 3:**
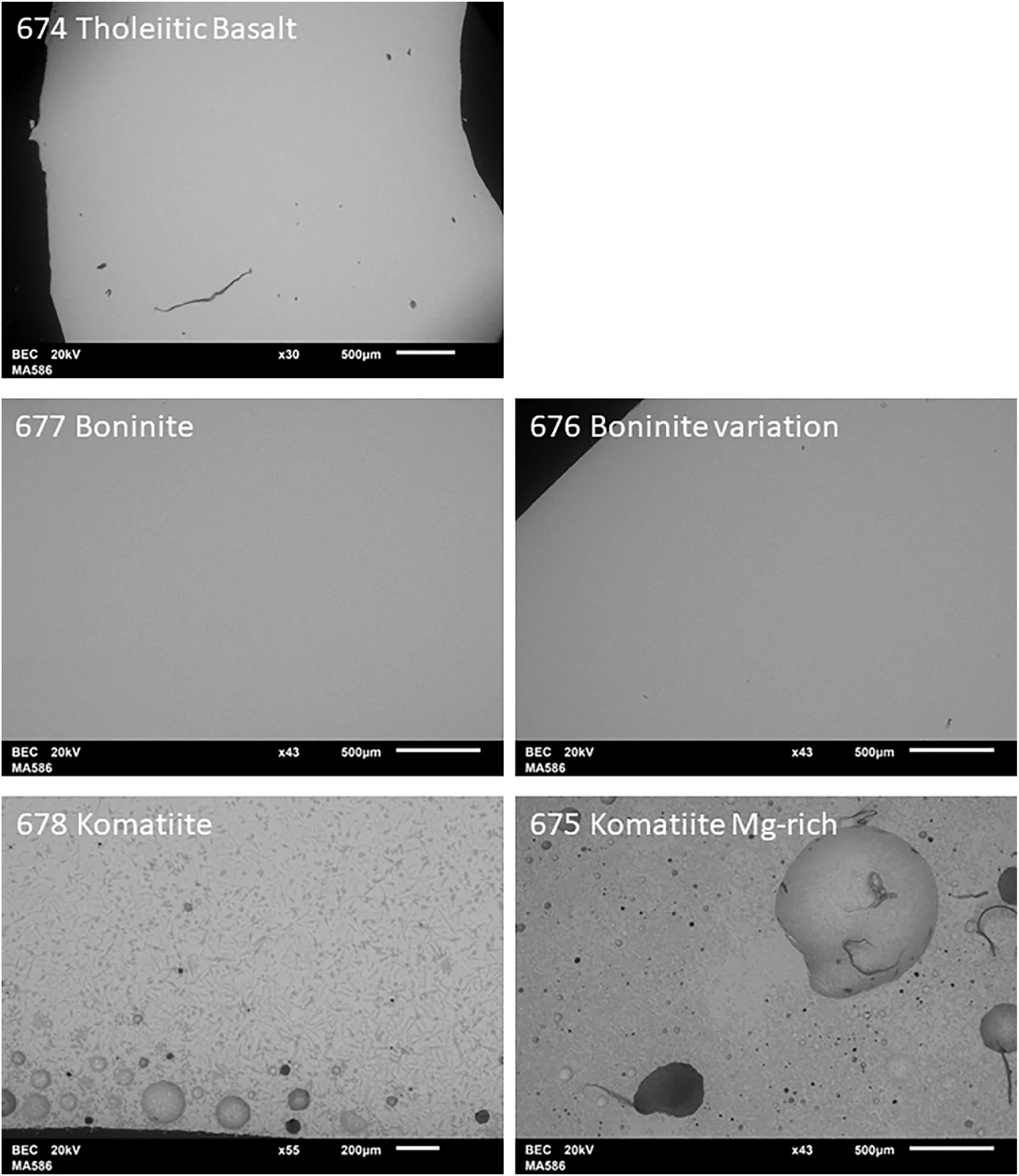
SEM backscattered electron images of polished blocks of the synthesized analogs. 678 Komatiite and 675 Komatiite Mg-rich contain abundant small, evenly distributed crystals within the glassy matrix, whereas the other samples are homogeneous glasses.

CF is a key feature in reflectance spectroscopy linked to the bulk composition. Plotting the CF of the samples against SiO_2_ (Fig. 4) [16] allows to classify the samples based on their spectroscopy. The boninite samples plot in the field of intermediate samples as well as for hermean analogs, while komatiites and tholeiitic basalts fall into the area of basic and basaltic rocks as well as lunar materials.

**Fig. 4:**
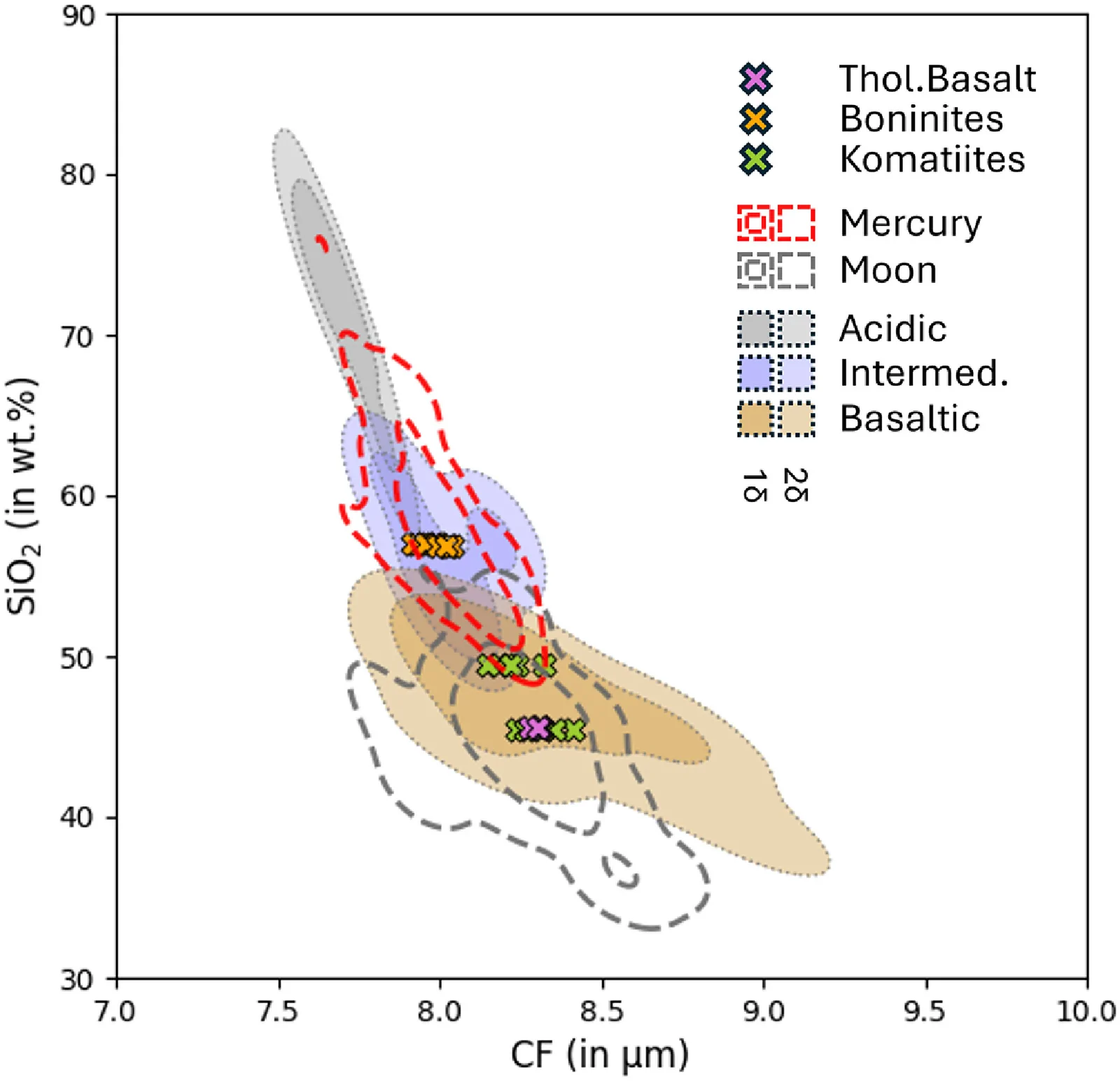
SiO_2_ (wt%) plotted against the CF position (in µm). Crosses are band positions from samples presented here: Green: Komatiites, Orange: Boninites, Pink: Tholeiitic Basalt. Dashed lines: 1 sigma (inner) 2 sigma (outer), red: Mercury, grey: Moon. Dotted lines: 1 sigma (inner) 2 sigma (outer), grey: acidic terrestrial rocks, blue: intermediate terrestrial rocks, brown: basaltic terrestrial rocks. Sources literature data: [13].

## 4 Experimental Design, Materials and Methods

### 4.1 Sample synthesis and preparation

Komatiites, boninites and basalts show wide ranges in chemistry. Covering all these potential compositions would be very difficult, so averages for the typical representatives of these rocks-types were applied [[5], [6], [7]].

The synthetic glasses were produced by mixing reagent-grade oxides (SiO₂, TiO₂, Al₂O₃, Fe₂O₃, MgO, MnO, Cr₂O₃, P_2_O_5_) and carbonates (CaCO₃, Na₂CO₃, K₂CO₃,)). For each composition 6 g of material was prepared. This is well above the minimum requirement needed for the size fractions (1 g), but also ensures that an aliquot of the homogenized powder is representative for the bulk material [e.g., 13].

The reagents were ground to a fine powder using an agate mortar with acetone. The final powders were then placed in Pt crucibles and decarbonized at 1000 °C. After decarbonization, the dried mixtures were melted in air at 1400 °C for 3 h using a high-temperature box furnace (Linn GmbH, Germany) located at the Institute for Mineralogy in Münster. The melts were then rapidly quenched by submerging the crucibles in water, achieving vitrification within seconds.

The tholeiitic glass was produced by completely melting a natural basaltic rock sample provided by the Institute of Mineralogy in Münster and subsequently quenched. The cooled glasses were crushed with a steel mortar under acetone. The powders were sieved into four grain size fractions (0 µm – 25 µm, 25 µm – 63 µm, 63 µm – 125 µm, 125 µm – 250 µm) using an automatic Retsch tap sieve.

### 4.2 Quality control and assurance and control (QC/AC)

A piece of each sample was embedded in epoxy resin, polished with a series of diamond pastes, and characterized using a JEOL 6610 scanning electron microscope (SEM) with an electron dispersive X-ray spectrometer (EDX) at the Institute of Mineralogy in Münster. Backscattered electron (BSE) images were acquired at an acceleration voltage of 20 kV. For SEM/EDX analyses, natural mineral standards were used (Astimex Ltd, Canada: olivine, clinopyroxene, orthopyroxene, chromite, feldspar). Measurements were taken from glassy parts avoiding crystallites. The detection limit for each element is 0.1 wt%.

For quality assurance (AC) only high quality commercially available reagent-grade chemical oxides and carbonates was used (Source: Alfa Aesar, Germany: SiO₂, TiO₂, Al₂O₃, Fe₂O₃, MgO, MnO, Cr₂O₃, P_2_O_5_, CaCO₃, Na₂CO₃, K₂CO₃,) as starting materials. Each mixture was weighed using a high precision microbalance (Kern GmbH, Germany).

Quality control (QC) of the final glasses was made using optical and scanning electron microscopy. in order to determine if the run product were homogenous. Here the images (Fig. 3) show very homogenous material for Basalt and Boninites. 678 Komatiite and 675 Komatiite Mg-rich exhibit small crystals in a predominantly glassy matrix, which are evenly distributed on a 500 µm^2^ scale, with no larger particles observed This ensures homogeneity of the sample when 6 *g* of synthesized material are homogenized and sample aliquots (1 g) are extracted for the sieving and spectral analyses.

### 4.3 Spectral measurements

The glass samples were analyzed by Fourier-transform infrared spectroscopy (FTIR). The glass powders were placed into aluminum cups with a diameter of 1 cm without compressing them to avoid inducing a preferred grain orientation. The samples were analyzed in the range from 2 µm to 18 µm at low pressure (10^−3^ bar) using in a Bruker Vertex 70v FTIR spectrometer at the IRIS laboratory in Münster in a bi-directional setup with an MCT Detector. For each grain-size fraction, 512 scans with a spectral resolution of 4 cm^−1^ were performed to increase the signal-to-noise ratio. Standard error is ±0.02 µm for each band position.

In addition, the samples were analyzed at different angles of incidence (i) and emergence (e) to simulate conditions on board an orbiting spacecraft. Scans were performed at angles 20° (incidence)/30° (emergence) by using a Bruker A513 multiple-angle reflectance accessory. The measurements were calibrated by using INFRAGOLD® as a diffuse reflectance standard. The background was automatically subtracted by ratioing, and the spectra have not been smoothed.

### 4.4 Data processing and presentation

The chemical and spectral data were processed using various Python open-source packages: [17,18,19]. Band position of the spectral features (Table 2) were obtained using a peak finding routine of the Python scipy.signal package [18]. The CF was identified by determine the position of lowest reflectance. Table 3

**Table 3:** Overview of data formats in the repository.

Name	Content	Format
ID Number_Sample Name_ Grain Size_ Temperature_ Pressure_ Geometry_	Mid-infrared diffuse reflectance spectra raw data for four size fractions per sample. first row: Wavenumbers (cm-1) second row: Wavelength (µm) third row: reflectance intensity	Text file (.txt)
ID_Number_Sample Name_Technique	Chemistry of samples obtained using SEM/EDX, in weight %. Technique: SEM = scanning Electron Microscopy with EDX.	Excel file (.xlsx)
ID_Number_Sample Name_Technique	SEM Backscattered electron images. Technique: BSE = Backscattered electron image	Figure (.jpg)

## Limitations

The spectra provided were obtained in diffuse reflectance. For applications with remote sensing data of hot surfaces, recalculation into emissivity using e.g., Kirchhoff’s law could be necessary [20].

## Ethics Statement

We have read and follow the ethical requirements for publication in Data in Brief and confirm that the current work does not involve human subjects, animal experiments, or any data collected from social media platforms.

## CRediT Author Statement

**Andreas Morlok:** Conceptualization, Methodology, Validation, Writing, Review & Editing, Supervision; **Oliver Burghardt:** Formal analysis, Investigation, Writing Original draft; **Stephan Klemme:** Conceptualization, Methodology, Validation, Writing, Review & Editing, Resources, Supervision **Iris Weber:** Methodology, Data Curation, Supervision; **Maximilian P. Reitze:** Supervision; **Harald Hiesinger:** Supervision, funding acquisition
